# Capability to identify and manage critical conditions: effects of an interprofessional training intervention

**DOI:** 10.1186/s12909-024-05567-z

**Published:** 2024-05-28

**Authors:** Ia Santesson, Carl Otto Schell, Petronella Bjurling-Sjöberg

**Affiliations:** 1 Centre for Clinical Research Sörmland, Uppsala University, Eskilstuna, 631 88 Sweden; 2 Department of Patient Safety, Region Sörmland, Eskilstuna, 631 88 Sweden; 3 Department of Global Public Health, Karolinska Institutet, Stockholm, 171 77 Sweden; 4 Department of Medicine, Nyköping Hospital, Nyköping, 611 88 Sweden; 5 Department of Public Health and Caring Sciences, Uppsala University, Uppsala, 752 37 Sweden

**Keywords:** Critical Illness, Critical Care, Quality of Care, Patient Safety, Interprofessional Education, Crew Resource Management

## Abstract

**Background:**

The burden of critical illness is a global issue. Healthcare systems often fail to provide essential emergency and critical care for deteriorating patients, and the optimal strategy for ensuring safe care is not fully known. This study aimed to explore the capability to identify and manage critical conditions and to evaluate how an interprofessional training intervention that included theory as well as high-fidelity simulation (proACT) in the short and long term affected the capability.

**Methods:**

A questionnaire study was performed. A cross-sectional survey of all in-hospital nurses and physicians in a Swedish region (n538) and a longitudinal cohort of participants entering the proACT course during a six-month period (n99) were included. Descriptive and comparative statistics were generated. Additionally, qualitative content analysis was performed for free text answers.

**Results:**

The findings demonstrated that the intervention improved the individual healthcare professionals’ competence with a sustained effect over time. The coverage of proACT trained staff increased from 13.2% to 26.5%, but no correlation was observed with workplace conditions that support safe care. Collaboration and workplace climate were perceived to be mainly positive, but for safer care, an overall need for improved competence and staffing was emphasized.

**Conclusions:**

The present study confirms previously identified issues and the need for improvements in the care of critically ill patients in general hospital wards. It supports the notion that a training intervention, such as proACT, can increase the capability to identify and manage patients with critical conditions. All healthcare professions increased the competence. Hence, more effort is needed to enable staff of all professions to participate in such training. Studies of interventions cover higher number of trained staff in the setting are warranted to clarify whether the training can also improve workplace conditions that support safe care of deteriorating and critically ill patients.

**Supplementary Information:**

The online version contains supplementary material available at 10.1186/s12909-024-05567-z.

## Background

Patients often present on the general hospital ward with signs of deterioration [1, 2]. The inability to recognize and respond appropriately to early signs of deterioration is a key patient safety issue [1–6]. Therefore, all frontline staff must be proactive and be able to provide essential emergency and critical care, including identifying patients with deteriorating conditions and taking appropriate action in a timely manner [1, 4, 7, 8]. For this, a hospital needs equipment, guidelines, routines, priority setting, and adequate competent staffing with workplace conditions that support safe care, including a workplace climate that promote reliable collaboration and communication [4].

“A deteriorating patient is one who moves from one clinical state to a worse clinical state which increases their individual risk of morbidity, including organ dysfunction, protracted hospital stay, disability, or death” [[9] p.1032–33].

To aid timely identification of deterioration and critical conditions, track and trigger systems, such as the National Early Warning Score (NEWS) [10, 11], are advocated [12, 13]. NEWS [10, 11] provides a systematic and standardized approach to assess vital parameters: respiratory rate, oxygen saturation (SpO_2_), systolic blood pressure, heart rate, level of consciousness and temperature, as well as supplied oxygen. The scores, based on the parameters, are aggregated to indicate a risk level, and an algorithm for escalation of care is provided, including when to seek help from a staff member with higher competence level [10, 11]. The NEWS has been proven to have the ability to discriminate patients at risk of cardiac arrest, unplanned ICU admission and death [12, 14–16]. However, education and caution in the use are recommended to avoid incorrect use of the scores [17].

To improve the prognosis of the patient, identification of critical illness needs to be accompanied by adequate actions, which require knowledge and competence of frontline staff [4, 18]. Furthermore, given the complexity of modern healthcare, reliable collaboration and communication are crucial for optimal outcomes [19]. A workplace climate with psychological safety that encourages everyone on the team to speak up if something feels wrong is vital for patient safety [20]. The staff thus, in addition to traditional knowledge and technical skills, also need non-technical skills about team interaction, and for this, crew resource management (CRM) is recommended [20]. CRM includes the following components: communication technique, leadership, teamwork, situational awareness and decision-making (including re-evaluation and seeking help). Applying CRM creates a climate that encourages an open attitude with the opportunity for everyone on the team, regardless of profession or title, to speak-up for patient safety if something feels wrong [20]. However, in practice, there is a variable performance on completing the safety behaviors prescribed in CRM [21].

To increase the competence in identifying and adequately acting on signs of deterioration and adopt CRM, several training programs have been launched, commonly utilizing blended learning, including traditional classroom education as well as simulation training [22]. One of these programs is the proACT course [23], developed by clinical healthcare professionals and managed by a non-profit association. This course provides interprofessional standardized training, utilizing a cascade principle for implementation, with main instructors training local instructors who in turn train clinical staff. The course aims to improve patient safety, and it is applied increasingly in Scandinavian hospitals. However, evidence of the outcome is lacking.

In this paper, we define competence as the quality of being competent, possessing required skills for identifying and managing patients with critical conditions, while capability refers to having the potential to apply the skills. Hence, the capability is affected by both the individual healthcare professional’s competence and the contextual condition in which the competence is supposed to be applied.

The aim of the present study was to explore the capability to identify and manage patients with critical conditions and to evaluate how an interprofessional training intervention that included theory as well as high-fidelity simulation (proACT) affected the capability. The specific research questions were as follows:

1. How does the training intervention affect different healthcare professionals’ competence in the short and long term regarding level of a) knowledge; b) familiarity and use of the NEWS; and c) self-confidence?
2. How do the staff perceive workplace conditions that support safe care in terms of a) collaboration and workplace climate; b) overall use of the NEWS; and c) overall patient safety; and d) are these workplace conditions affected by the proportion of the healthcare professionals in the unit who had completed the training intervention (proACT coverage)?

## Methods

A questionnaire study with a quasi-experimental design was performed, combining a cross-sectional survey of all in-hospital nurses and physicians in a Swedish region and a longitudinal cohort of participants entering the proACT course during a six-month period.

The purpose of the cross-sectional survey was to obtain data from a variety of respondents in different wards, and thereby be able to explore the capability to identify and manage critical conditions across an entire healthcare organization. Additional purpose was to explore eventual differences in competence between respondents with completed proACT training and those with no such training, and eventual differences regarding workplace conditions between units with different levels of proACT coverage. The purpose of the longitudinal cohort was to complement the cross-sectional survey and enable exploration of how the proACT training affected the responders’ competence over time. Finally, the purpose of combining findings from the cross-sectional survey and the longitudinal cohort was to strengthen the ability to make accurate conclusions of how the training intervention affected the capability.

### Setting and intervention

The study took place from October 2018 to December 2019 in a public healthcare organization with three somatic hospitals (~ 300, 200 and 70 beds). The hospital wards were staffed by registered nurses (RNs) and assistant nurses (ANs) around-the-clock. Physicians conducted daily rounds and were on duty the rest of the time for consultation and on demand attendance.

Within the organization, the significance of the problem of suboptimal care of deteriorating patients had been highlighted by several stakeholders. To increase patient safety and optimize resource utilization, the healthcare board in 2018 initiated a three-piece regional investment including mandatory use of NEWS [10, 11], establishing Clinical Training Centers (CTCs), and implementing mandatory proACT [23] training for all RNs, ANs and physicians in adult somatic care.

Information about the NEWS [10, 11] was provided in guidelines, the internal web, assembly hall meetings and e-mails. All patients should be assessed according to the NEWS algorithm, which was a task mainly assigned to ANs, but also RNs could do the assessment. Both ANs and RNs had responsibility to alert for deterioration. Physicians were supposed to inquire for the NEWS assessment and use it when seeking help from a provider with higher competence level. All professions were supposed to perform basic life-saving actions, although prescription of pharmaceuticals was restricted to the physicians and administration of pharmaceuticals restricted to the RNs. The established CTCs provided opportunities for high-fidelity simulation with guidance from trained instructors. The CTC administration coordinated the proACT training resources, while responsibility to provide local instructors and to select and enable staff to participate in the training was left to the management of each clinical department.

In the proACT training, a systematic approach to evaluate and re-evaluate a patient’s state and basic life-saving actions was provided based on the ABCDEF-acronym (Airway, Breathing, Circulation, Disability, Exposure and Further care), NEWS [10, 11], CRM [20], communication techniques and basic medical ethics. In accordance with the structure of the standardized course [23], all participants, regardless of their profession, started the program by reading a theoretical textbook [13], which also included practical examples. Thereafter, they completed the proACT digital exercises, including interactive reflections and a self-test [23]. Finally, they participated in a one-day training. To participate they had to testify that they had read the textbook and present a certificate of completed digital exercises. The one-day training (8 h) included a half-day class-room seminar and a half-day interprofessional high-fidelity simulations (4 × 45 min) with reflection, including training to identify deterioration, communicate the situation, collaborate with the healthcare team and take adequate actions. Each simulation session included 10 min pre-briefing to describe the prerequisites and the case, 15 min simulation of a clinical situation and 20 min debriefing. The debriefing included three phases. First, in the descriptive phase, each participant described the situation and actions from their own perspective. Next, in the analysis phase, the participants were encouraged to reflect upon the sequence of events and identify adequate actions, shortcomings and the causes. Finally, in the applicability phase, lessons for next simulation session and for clinical practice were summarized.

### Recruitment and data collection

**Fig. 1:**
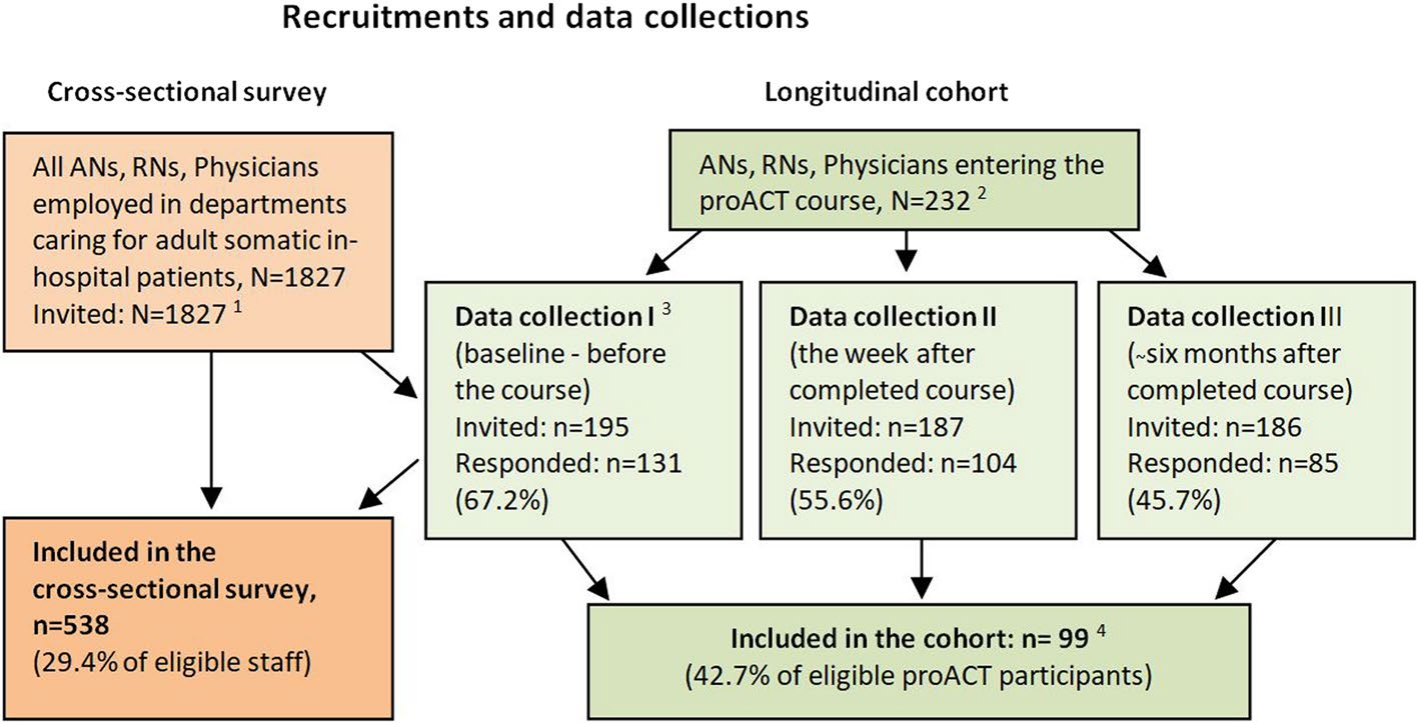
Flowchart of recruitment and data collection in the cross-sectional survey and in the longitudinal cohort of participants entering the proACT program during the study period

Recruitment and data collection were conducted in two phases using the same questionnaire (see Fig. 1).

First, a cross-sectional survey was performed, to which all ANs, RNs and physicians employed in departments caring for adult somatic in-hospital patients were invited. Employees exclusively working in the operating theatre, obstetrical ward or emergency unit were excluded. All who met the inclusion criteria (811 ANs, 542 RNs and 474 physicians; *N* = 1827) were approached by e-mail, including information about the study, voluntary participation, and a web link to a study-specific questionnaire.

Second, a longitudinal cohort study was performed, to which all ANs, RNs and physicians who entered the proACT training during a six-month period from the survey were invited (105 ANs, 100 RNs and 27 physicians; *N* = 232). These course participants were informed of the study by e-mail and asked to answer the questionnaire: I) before starting the course, if they had not already answered the survey when invited to the cross-sectional study; II) the week after the training session; and III) six months later.

All respondents who completed the questionnaire were included in the survey. Participants who completed the questionnaire at least twice were included in the cohort sample.

### Questionnaire

The study-specific web-based questionnaire was developed by a local group of training coordinators (RNs from the CTC), a patient safety coordinator, an anesthesiologist and a resident physician. Pilot testing, using the think-aloud technique [24], rendered satisfying face validity after some minor language clarifications.

The questionnaire included an 18-question test to reflect the knowledge of how to identify and manage critical conditions based on the proACT textbook [13] and the National Handbook for Healthcare [11]. Each question had one correct and 2–3 incorrect statements and the possibility to mark “I do not know”. Furthermore, the questionnaire included two questions about familiarity and use of the NEWS (3 options closed response alternative); ten statements regarding self-confidence, perception of collaboration, workplace climate, overall use of the NEWS and overall patient safety (answered on 5-point Likert scales); background characteristics (profession, professional experience, workplace, gender, age); and whether the respondent had ever completed a proACT training or other similar course. Finally, space was given for free text comments regarding capability to identify and manage patients with critical conditions in their workplace.

### Analysis

Quantitative data were processed to generate descriptive and comparative statistics using SPSS statistics (SPSS 29.0.0 IBM Corp., Armok, NY, USA). T tests for equality of means (2-tailed) and paired T tests for equality of means (2-tailed) were used for cross-sectional and cohort data, respectively. The interaction effect was analyzed by univariate analysis of variance (ANOVA), with profession and no proACT/Completed proACT as explanatory variables. ANOVA was also used in the subgroup analysis, with profession and time since completing the course as explanatory variables. The Mann‒Whitney U test (2-tailed) and Wilcoxon signed rank test (2-tailed) were used for ordinal level data. For the cohort data, three comparisons of the responders’ answers from the different data collections were analyzed: I versus II; II versus III; I versus III. The significance level was set to *p* 0.05.

To evaluate whether workplace conditions that support safe care were affected by the proportion of staff that had completed proACT (proACT coverage), a separate multivariable logistic regression was performed. Each answer about the workplace conditions was dichotomized into either”positive” or”neutral/negative” (including 4–5 or 1–3 on the Likert scale, respectively) and used as the dependent variable. The proportion of proACT-trained staff of each profession in the respondent’s workplace (unit) at the time the questionnaire was answered was used as an explanatory variable. Due to the high number of analyses, Bonferroni correction was used for hypothesis testing, which gave a significance level of *p* 0.001.

Free text answers were analyzed using inductive qualitative content analysis [25], including three steps. First, meaning units related to the research questions were identified, condensed and coded with focus on the manifest content. Second, the codes were compared and grouped into main and sub-categories based on similarities and differences. Examples of this analysis process are provided in Table 1. Finally, two overall themes of comments were identified that both related to the main categories, and a storyline that described the content was written.

**Table 1:** Example of the analysis process of the free text comments

Free text comments	Codes	Sub-categories	Main categories
You need to have clear communication between colleagues for patient safety and better care. (comment from a physician)	Important to communicate in the care team	Team communication	Communication and collaboration
It is important to report deviating parameters and the general condition to the RN, observe if the patient is deteriorating and alert the RN (comments from an AN)	Assessing the patients status Reacting on signs of deterioration Reporting to higher competence	Assessing and reacting	Competence and actions
When there is a lack of staff and often overcrowding, it is difficult to have time to make these assessments optimally (comment from a RN)	Insufficient staffing hinders optimal surveillance	Staffing	Staffing and organisation

*Abbreviations*: *AN* Assistant nurses, *RN* Registered nurses

## Results

### Characteristics of the respondents and proACT coverage

In total, 538 respondents participated in the cross-sectional survey (29.4% of eligible healthcare professionals; 27.1%, 40.0%, 13.5% of the ANs, RNs and physicians, respectively). The longitudinal cohort included 99 respondents who had answered the questionnaire twice or three times (42.7% of eligible proACT participants; 40.0%, 43.0%, 51.9% of participating ANs, RNs and physicians, respectively) (Fig. 1). The characteristics of the respondents are displayed in Table 2. Concordant with the distribution of employees, 54.0% of the respondents worked in the largest hospital, 29.0% in a mid-sized hospital and 17.0% in the smallest hospital.

**Table 2:** Characteristics of the respondents in the cross-sectional survey and the longitudinal cohort

**Respondents**		**Cross-sectional survey *n* = 538**		**Longitudinal cohort** ***n* = 99**	
		n	(%)	n	(%)
Gender	Female	451	(83.8)	84	(84.8)
	Male	82	(15.2)	15	(15.2)
	Other/missing	5	(0.93)	-	
Age (years)	≤ 29	102	(19.0)	21	(21.2)
	30–39	145	(27.0)	22	(22.2)
	40–49	126	(23.4)	25	(25.3)
	50–59	116	(21.6)	25	(25.3)
	≥ 60	49	(9.10)	6	(6.1)
Profession	Assistant nurse	220	(40.9)	42	(42.4)
	Registered nurse	217	(40.3)	43	(43.4)
	Physician	101	(18.8)	14	(14.1)
Years in the profession	< 2	105	(19.5)	29	(29.3)
	2–5	11	(20.6)	17	(17.2)
	6–10	86	(16.0)	16	(16.2)
	11–20	101	(18.8)	12	(12.1)
	≥ 21	135	(25.1)	25	(25.3)
Completed a proACT course	No	244	(45.3)		
	Yes^a^	270	(50.2)		
	Only read the book^b^	14	(2.60)		
	Not equivalent course^b^	10	(1.86)		
Months since completing a proACT course	< 1	57	(21.3)		
	1–6	66	(24.6)		
	7–12	115	(42.9)		
	> 12	30	(11.2)		

Notes: ^a^Includes also responders who completed the previously provided ALERT course (acronym for Acute Life-threatening Events—Recognition and Treatment), which was judged as equivalent to proACT

^b^Due to the small sample size, these responders were not included in the comparative analyses

The proACT coverage increased from 13.2% to 26.5% during the study period, but the proportion of trained staff differed between professions and between care units (hospital wards/department). A majority of the units lacked proACT^−^trained physicians, while the coverage was higher for ANs and RNs at the start as well as the end of the study (Table 3).

**Table 3:** Coverage of proACT-trained staff

**Proportion of proACT trained staff, %**^**1**^															
	**Start of the study**							**End of the study**							
	**Overall in the region**		**Mean per unit**		**Min–max per unit**			**Overall in the region**		**Mean per unit**			**Min–max per unit**		
ANs	16.5		16.5		0–71.4			31.2		30.9			0–100		
RNs	19.6		21.5		0–100			38.4		37.5			0–100		
Physicians	0.41		1.04		0–16.7			5.92		5.10			0–29.0		
Total	13.2							26.5							
**Number (%) of units with different proportion of proACT trained staff**^a^															
	**Start of the study**							**End of the study**							**Total**
	**0%**	**1-25%**		**26-50%**		**51-75%**	**> 75%**	**0%**	**1-25%**		**26-50%**	**51–75%**		**> 75%**	**number of units**
ANs	7 (26.9)	14 (53.8)		2 (7.7)		3 (11.5)	-	2 (7.7)	11 (42.3)		7 (26.9)	4 (15.4)		2 (7.7)	26 (100)
RNs	7 (26.9)	11 (42.3)		5 (19.2)		1 (3.8)	2 (7.7)	4 (15.4)	6 (23.1)		8 (30.8)	5 (19.2)		3 (11.5)	26 (100)
Physicians	15 (93.8)	1 (6.2)		-		-	-	12 (75.0)	3 (18.8)		1 (6.2)	-		-	16 (100)

Notes: Overall proportion and number of units with different proportions of staff who had completed the training at the start and end of the study

^a^For ANs and RNs, a unit is a hospital ward (*n* = 26), and for physicians, a unit is a hospital department (*n* = 16), as physicians work in several wards (thereby no total per unit)

*Abbreviations*: *AN* Assistant nurses, *RN*, Registered nurses

### The healthcare professionals’ competence

#### Knowledge of how to identify and manage patients with critical conditions

**Fig. 2:**
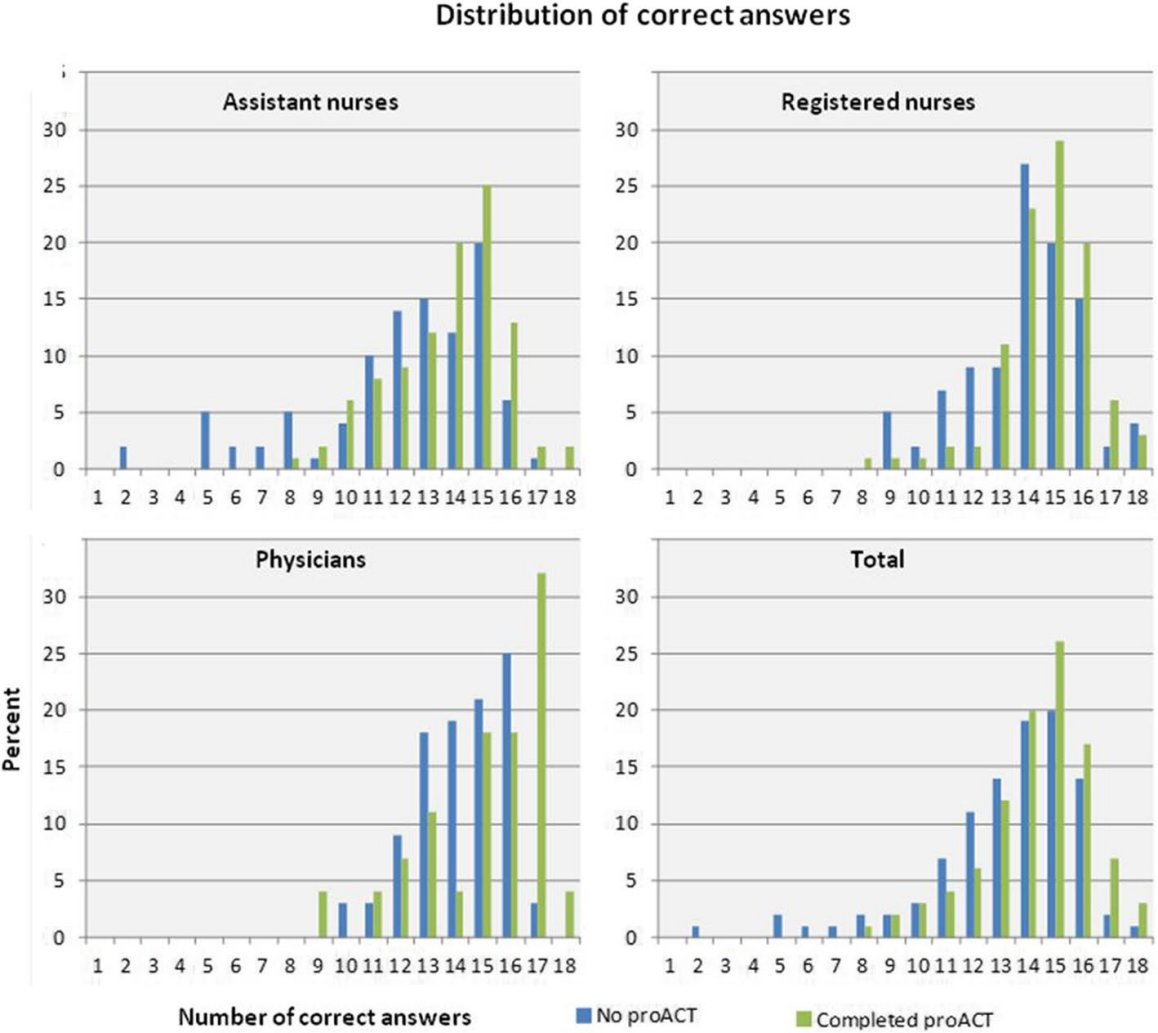
Distribution of correct answers in the 18-question test reflecting knowledge of how to identify and manage patients with critical conditions. Comparing responders in the cross-sectional survey with no proACT training versus those with completed proACT training

Knowledge of how to identify and manage critical conditions was assessed by the number of correct answers in the 18-question test. In the cross-sectional survey, knowledge was higher in the group that had proACT training than in the group that did not (mean score 14.30 vs. 13.27, *p* < 0.001) (Table 4). In both groups, there was a significant difference in the mean score between the professions (ANs < RNs < physicians, *p* < 0.001), but all professions significantly benefited from the training. The distribution of correct answers for each profession is displayed in Fig. 2. Subgroup analyses regarding time since proACT training revealed the highest mean scores in the group of respondents with newly completed training (< 1 month ago 14.68, SD 1.87; 1–6 months 14.09, SD 2.09; 7–12 months 14.50, SD 1.81 and; > 12 months 13.43, SD 2.08; *p*.004). In the longitudinal cohort, knowledge significantly increased after the training, from a mean score of 12.72 before to 15.31 the week after (*p* < 0.001) and sustained improvement six months later (mean score 14.37, *p* < 0.001) (Table 4).

**Table 4:** Knowledge of how to identify and manage patients with critical conditions, displayed by number of correct answers in the 18 question test^1^

**Cross-sectional survey data**																					
	**No proACT**						**Completed proACT**						**No proACT vs. Completed proACT**								
	**n**	**mean**	**SD**	**min**	**-**	**max**	**n**	**mean**	**SD**	**min**	**-**	**max**	*P*								
ANs	95	12.04	3.31	2	-	17	119	13.71	2.06	8	-	18	< .001^↑^								
RNs	81	13.89	2.09	9	-	18	123	14.69	1.66	8	-	18	.003^↑^								
Physicians	68	14.24	1.65	10	-	17	28	15.11	2.22	9	-	18	.037^↑^								
Total	244	13.27	2.72	2	-	18	270	14.30	1.97	8	-	18	< .001^↑^								
**Longitudinal cohort data**^**2**^																					
	**I—Before proACT**						**II—Week after**						**III—Six month later**						**I vs. II**	**II vs. III**	**I vs. III**
	**n**	**mean**	**SD**	**min**	**-**	**max**	**n**	**mean**	**SD**	**min**	**-**	**max**	**n**	**mean**	**SD**	**min**	-	**max**	*P*	*P*	*P*
ANs	38	11.89	3.12	6	-	18	37	14.78	1.95	10	-	18	32	13.47	1.65	9	-	16	< .001^↑^	.010^↓^	*.002*^↑^
RNs	36	13.28	1.92	9	-	18	40	15.33	2.29	5	-	18	30	14.77	1.89	8	-	18	< .001^↑^	.211	< .001^↑^
Physicians	14	13.50	1.95	8	-	15	10	15.40	1.84	11	-	17	11	15.91	1.51	13	-	18	.055	.231	.003^↑^
Total	88	12.72	2.59	6	-	18	87	15.31	2.10	5	-	18	73	14.37	1.93	8	-	18	< .001^↑^	.037^↓^	< .001^↑^

Notes: First, the results from the cross-sectional survey compared responders without proACT training versus those with completed proACT training. Second, the results from the longitudinal cohort compared the responses of individuals before versus the week after the proACT course (I vs. II); the week after versus six months later (II vs. III); and before versus six months later (I vs. III). ^1)^ 18-question test reflecting the knowledge of how to act to identify and manage critical conditions. ^2)^ Descriptive statistics are displayed for all respondents in each dataset, although internal dropout meant varied number of respondents included in respective comparison. ^↑)^ Significant positive difference (*p* ≤ .05). ^↓)^ Significant negative difference (*p* ≤ .05)

*Abbreviations*: *AN* Assistant nurses, *RN* Registered nurses

### Familiarity with and use of the NEWS

Familiarity and use of the NEWS were higher in the cross-sectional survey in the group that had proACT training than in the group that had not (*p* < 0.001 for both questions). In the longitudinal cohort, respondents who knew the NEWS well increased from 71.6% to 95.4% after the training, with sustained improvement six months later (90.4%, *p*.006). Daily use increased from 34.1% to 51.7% after the training, with sustained improvement six months later (56.2%, *p*.002). The increased use was most evident for the physicians (from 14.3% to 54.5.0%, *p*.020) (Table 5).

**Table 5:** Familiarity and use of the National Early Warning Score (NEWS) routine

**Familiarity and use of the NEWS routine**^**1**^											
**Cross-sectional survey data**											
		**No proACT**		**Completed proACT**					**No proACT vs. Completed proACT**		
		**Total n**	**Yes %**	**Total n**		**Yes %**			*p*		
I am familiar with the NEWS routine^2^	ANs	93	72.0	118		97.5			< .001^↑^		
	RNs	80	77.5	113		91.2			.004^↑^		
	Physicians	64	56.3	28		82.1			.014^↑^		
	Total	237	69.6	259		93.1			< .001^↑^		
		Total n	Daily %	Total n		Daily %			*p*		
I use the NEWS routine^3^	ANs	93	30.1	118		65.3			< .001^↑^		
	RNs	80	36.3	113		61.1			< .001^↑^		
	Physicians	64	32.8	28		50.0			.061		
	Total	237	32.9	259		61.8			< .001^↑^		
**Longitudinal cohort data**^3^											
		**I—Before proACT**		**II—Week after**			**III—Six month later**		**I vs. II**	**II vs. III**	**I vs. III**
		**Total n**	**Yes %**	**Total n**	**Yes %**		**Total n**	**Yes %**	*p*	*p*	*p*
I am familiar with the NEWS routine^2^	ANs	38	78.9	37	100		32	93.8	.007^↑^	.157	.014^↑^
	RNs	36	72.2	40	92.5		30	90.0	.014^↑^	.564	.132
	Physicians	14	50.0	10	90.0		11	81.8	.046^↑^	.317	.317
	Total	88	71.6	87	95.4		73	90.4	< .001^↑^	.317	.006^↑^
		Total n	Daily %	Total n	Daily %		Total n	Daily %	*p*	*p*	*p*
I use the NEWS routine^3^	ANs	38	34.2	37	43.2		32	59.4	.109	.058	.033^↑^
	RNs	36	41.7	40	62.5		30	53.3	.285	.999	.366
	Physicians	14	14.3	10	40.0		11	54.5	.034^↑^	.157	.020^↑^
	Total	88	34.1	87	51.7		73	56.2	.009^↑^	.102	.002^↑^

Notes: First, the results from the cross-sectional survey compared responders without proACT training versus those with completed proACT training. Second, the results from the longitudinal cohort compared the responses of individuals before versus the week after the proACT course (I vs. II); the week after versus six months later (II vs. III); and before versus six months later (I vs. III).^1)^ Three options closed response alternative; the table displays the most positive response. Additional file 1 displays the full distribution of responses. ^2)^ Response options: “No” = I have previously not heard of the NEWS routine, “Unsure” = I know that the routine exists but is unsure of the content, Yes” = I know about the routine and is aware of the content and what it means in my work. ^3)^ Response options: “Never”, “Sometimes” or “Daily”. ^3)^ Descriptive statistics are displayed for all respondents in each dataset, although internal dropout meant varied number of respondents included in respective comparison. ^↑)^ Significant positive difference (*p* ≤ .05). ^↓)^ Significant negative difference (*p* ≤ .05)

*Abbreviations*: *AN* Assistant nurses, *RN* Registered nurses

### Self-confidence

Levels of self-confidence are displayed in Table 6. In the survey, proACT trained staff compared with the group without such training, to a higher degree agreed (totally or partly) on self-confidence to identify signs (94.9% vs. 90.3%), initially treat (90.3% vs. 78.1%), communicate (93.1% vs. 86.0%) and speak-up (98.1% vs. 92.4%), although significant differences were found only regarding self-confidence of initial treatment (*p*.002). Subgroup analyses revealed significant differences between trained vs. not trained ANs in all items (self-confidence to identify signs 93.2% vs. 86.0%, *p*.006; initially treat 85.6% vs. 64.5%, *p* < 0.001; communicate 91.6% vs. 77.4%, *p*.003; speak-up 99.2% vs. 93.6%, *p*.009). Between the groups of physicians, there was a significant difference regarding the self-confidence to identify signs (100% vs. 93.7%, *p*.026). Between the groups of RNs, no significant differences were found. The analysis regarding when the training was completed did not reveal any significant difference in self-confidence in the total sample or the separate professions.

**Table 6:** Level of self-confidence

**Lever of self-confidence**^**1**^																			
**Cross-sectional survey data**																			
		**No proACT**					**Completed proACT**					**No vs. Completed proACT**							
		**n**	**Pos.%**	**Md**	**IQR**	**Min–max**	**n**	**Pos.%**	**Md**	**IQR**	**Min–max**	*p*							
I am confident to identify signs	ANs	93	86.0	4	1	1–5	118	93.2	4	1	3–5	.006^↑^							
	RNs	80	92.6	5	1	1–5	113	95.6	4	1	2–5	.359							
	Physicians	64	93.7	4	1	2–5	28	100	5	1	4–5	.026^↑^							
	Total	237	90.3	4	1	1–5	259	94.9	4	1	1–5	.072							
I am confident of initial treatment	ANs	93	64.5	4	1	1–5	118	85.6	4	0	1–5	< .001^↑^							
	RNs	80	83.8	5	1	2–5	113	94.7	5	1	2–5	.524							
	Physicians	64	90.6	4	1	2–5	28	92.9	5	1	3–5	.098							
	Total	237	78.1	4	1	1–5	259	90.3	4	1	1–5	.002^↑^							
I am confident to communicate the condition	ANs	93	77.4	4	1	1–5	118	91.6	4	1	2–5	.003^↑^							
	RNs	80	91.3	5	1	2–5	113	94.7	5	1	2–5	.603							
	Physicians	64	92.2	5	1	2–5	28	92.8	5	1	3–5	.598							
	Total	237	86.0	4	1	1–5	259	93.1	5	1	2–5	.117							
I have courage to speak-up	ANs	93	93.6	5	1	1–5	118	99.2	5	1	3–5	.009^↑^							
	RNs	80	92.6	5	0	2–5	113	96.5	5	1	2–5	.497							
	Physicians	64	90.7	5	1	1–5	28	100	5	1	4–5	.635							
	Total	237	92.4	5	1	1–5	259	98.1	5	1	1–5	.053							
**Longitudinal cohort data**^2^																			
		**I—Before proACT**					**II- Week after**					**III—Six month later**					**I vs. II**	**II vs. III**	**I vs. III**
		**n**	**Pos.%**	**Md**	**IQR**	**Min–max**	**n**	**Pos.%**	**Md**	**IQR**	**Min–max**	**n**	**Pos.%**	**Md**	**IQR**	**Min–max**	*p*	*p*	*p*
I am confident to identify signs	ANs	38	68.5	4	1	3–5	37	94.9	4	1	3–5	32	93.7	4	1	3–5	.001^↑^	.035^↓^	.003^↑^
	RNs	36	85.5	4	1	1–5	40	100	4	1	4–5	30	93.4	4	1	2–5	.003^↑^	.589	.360
	Physicians	14	100	4	1	4–5	10	100	4.5	1	4–5	11	100	5	1	4–5	.317	.564	.046^↑^
	Total	88	78.4	4	0.75	1–5	87	97.7	4	1	3–5	73	94.5	4	1	2–5	< .001^↑^	.283	.001^↑^
I am confident of initial treatment	ANs	38	57.9	4	1	1–5	37	88.8	4	0	3–5	32	91.6	4	0	3–5	< .001^↑^	.405	.001^↑^
	RNs	36	72.3	4	1	2–5	40	100	4.5	1	4–5	30	96.7	4	1	3–5	< .001^↑^	.405	.019^↑^
	Physicians	14	85.7	4	0.25	3–5	10	90.0	4	1	3–5	11	90.9	5	1	3–5	.180	.999	.025^↑^
	Total	88	68.2	4	1	1–5	87	94.3	4	1	3–5	73	93.2	4	1	3–5	< .001^↑^	.999	< .001^↑^
I am confident to communicate the condition	ANs	38	78.9	4	1	2–5	37	94.6	4	1	3–5	32	90.6	5	1	3–5	< .001^↑^	.366	.007^↑^
	RNs	36	75.0	4	1.75	1–5	40	100	5	1	4–5	30	90.0	4	1	2–5	< .001^↑^	.366	.166
	Physicians	14	85.7	4	1	3–5	10	90	5	1	3–5	11	90.9	4	1	3–5	.025^↑^	.414	.589
	Total	88	78.4	4	1	1–5	87	96.6	5	1	3–5	73	90.4	4	1	2–5	< .001^↑^	.705	.004^↑^
I have courage to speak-up	ANs	38	97.4	5	1	3–5	37	94.6	5	1	3–5	32	100	5	0.75	4–5	.282	.480	.285
	RNs	36	97.2	5	1	1–5	40	100	5	1	4–5	30	96.7	5	0.25	3–5	.029^↑^	.405	.059
	Physicians	14	92.9	5	1	3–5	10	90	4	1.25	3–5	11	100	5	1	4–5	.180	.414	.655
	Total	88	96.6	5	1	1–5	87	95.4	5	1	3–5	73	98.6	5	0	3–5	.096	.182	.117

Notes: First, the results from the cross-sectional survey compared responders without proACT training versus those with completed proACT training. Second, the results from the longitudinal cohort compared the responses of individuals before versus one week after the proACT course (I vs. II); one week after versus six months later (II vs. III); and before versus six months later (I vs. III).^1)^ 5-point Likert scale: Totally disagree = 1 to Totally agree = 5. Table displays % positive responses (4–5 on the Likert scale). Additional file 2 displays the full distribution of responses. ^2)^ Descriptive statistics are displayed for all respondents in each dataset, although internal dropout meant varied number of respondents included in respective comparison. ^↑)^ Significant positive difference (*p* ≤ .05). ^↓)^ Significant negative difference (*p* ≤ .05)

*Abbreviations*: *AN* Assistant nurses, *RN* Registered nurses, *Md* Median, *IQR* Interquartile range

In the cohort, the level of self-confidence increased from before the training until the week after, with a total proportion that agreed (totally or partly) about self-confidence to identify signs of critical conditions increasing from 78.4% before to 97.7%, *p* < 0.001, initial treatment from 68.2% to 94.3%,* p* < 0.001, and communication from 78.4% to 96.6%,* p* < 0.001. Six months later, the significant improvements were sustained, except for courage to speak-up, which did not increase significantly. Subgroup analysis revealed a significant increase for ANs regarding all items besides courage to speak-up, for RNs regarding treatment, and for physicians regarding identify signs and initial treatment (Table 6).

### Workplace conditions that support safe care

No significant correlation was found between workplace conditions that support safe care and the proACT coverage (*p* > 0.001 for all items, Bonferroni-adjusted p value). A large majority of the respondents in the cross-sectional survey perceived that collaboration worked well or very well with ANs (92.6%), RNs (96.9%) and physicians (85.2%) when a patient had signs of developing a critical condition. A majority also totally or partly agreed with the statements that they were well treated when they needed help (93.3%), that most staff used NEWS (64.9%) and that they would feel safe if a loved one was cared for in their workplace (80.5%). Additionally, the subgroup analysis of each profession revealed a majority of positive perceptions regarding the workplace conditions (Table 7).

**Table 7:** Workplace conditions as perceived by the total sample in the cross-sectional survey

	**Workplace conditions**^**1**^					
**Question**	**Responders**		**Positive, %**	**Md**	**IQR**	**Min–max**
Collaboration with assistant nurses	ANs	(*n* = 211)	96.2	5	1	2–5
	RNs	(*n* = 206)	91.7	5	1	1–5
	Physicians	(*n* = 98)	86.8	4	1	1–5
	Total	(*n* = 515)	92.6	5	1	1–5
Collaboration with registered nurses	ANs	(*n *= 211)	98.1	5	1	3–5
	RNs	(*n* = 206)	98.1	5	1	3–5
	Physicians	(*n* = 98)	91.9	4	1	1–5
	Total	(*n* = 515)	96.9	4	1	1–5
Collaboration with physicians works	ANs	(*n* = 211)	83.4	4	1	1–5
	RNs	(*n* = 206)	84.5	4	1	1–5
	Physicians	(*n* = 98)	90.8	5	1	1–5
	Total	(*n* = 515)	85.2	4	1	1–5
I meet positive response when I need help	ANs	(*n* = 211)	95.2	5	1	1–5
	RNs	(*n* = 206)	92.7	5	1	1–5
	Physicians	(*n* = 98)	89.8	4	1	2–5
	Total	(*n* = 515)	93.3	5	1	1–5
Most staff use the NEWS	ANs	(*n* = 211)	75.8	4	1	1–5
	RNs	(*n* = 206)	57.3	4	1	1–5
	Physicians	(*n* = 98)	57.1	4	1	1–5
	Total	(*n* = 515)	64.9	4	1	1–5
I would feel safe if a loved one was cared for at my workplace	ANs	(*n *= 211)	83.4	4	1	1–5
	RNs	(*n* = 206)	80.1	4	1	1–5
	Physicians	(*n *= 98)	77.5	4	1	1–5
	Total	(*n *= 515)	80.5	4	1	1–5

Notes: ^1)^ All questions answered on a 5-point Likert scale with the most negative response as 1 and the most positive response as 5. “Positive” includes responses of 4–5 on the Likert scale. Additional file 3 displays the full distribution of responses

*Abbreviations*: *AN* Assistant nurse, *RN* registered nurse, *NEWS* National Early Warning Score, *Md* Median, *IQR* Interquartile range

**Fig. 3:**
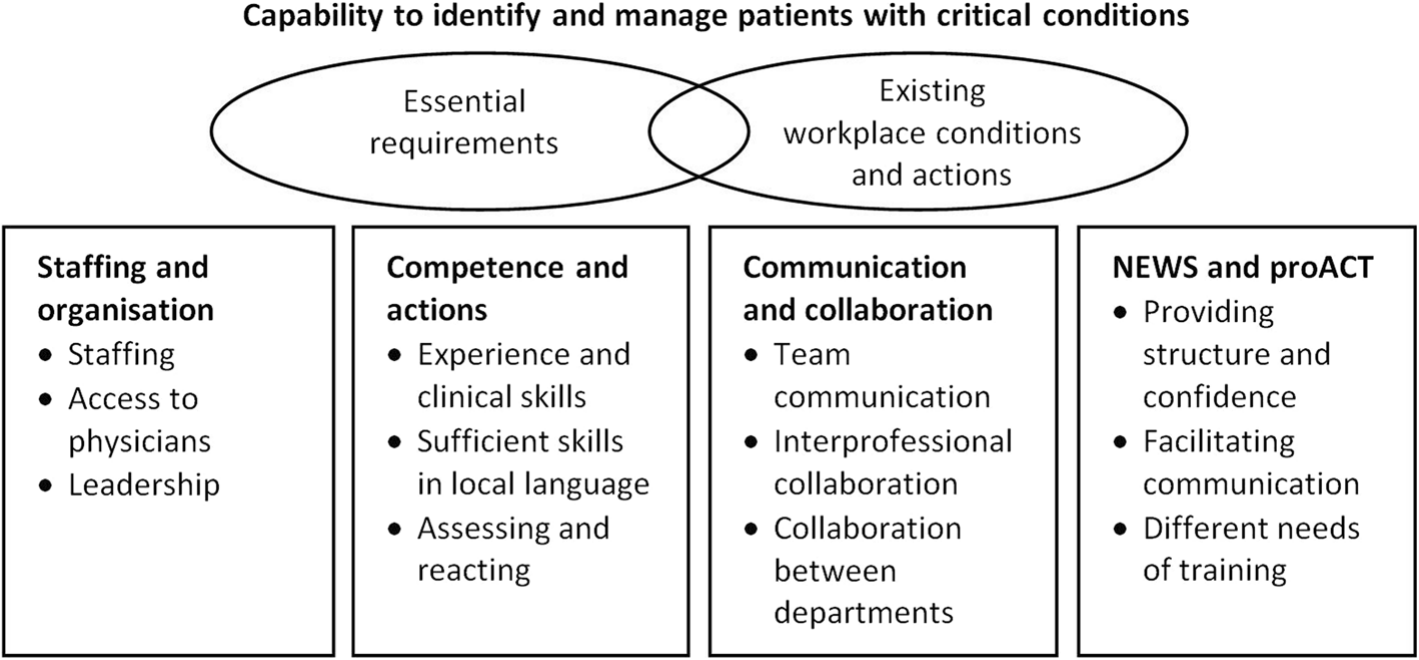
Results from the content analysis of the free text comments regarding capability to identify and manage patients with critical conditions. Boxes indicate categories, with subcategories. Ovals indicate the two overall themes, both related to the identified categories

In total, 57 free text comments regarding capability were received. The content analysis of the comments rendered four main categories with sub categories and two overall themes. Together they conveyed the responders’ perceptions of essential requirements for optimal capability to identify and manage patients with critical conditions, and their perception of existing workplace conditions and actions (see Fig. 3).

Essential requirements for optimal capability included adequate staffing, access to physicians, and leadership. Experiences, clinical skills and sufficient skills in local language were expressed as important for optimal competence. Actions and manners, including properly assessing and reacting, communicating and collaborating, were expressed as important. Additionally, interprofessional collaboration and willingness to help were expressed as essential.

Experiences of existing workplace conditions and actions varied, and the essential requirements for optimal capability were perceived to be fulfilled to different degrees. Overcrowding and insufficient staffing were expressed to sometimes hinder optimal monitoring of the patients, and insufficient access to physicians led to a feeling of exposure, especially at night. Some temporary staff with insufficient skills in the local language entailed a communication barrier. Additionally, although interprofessional collaboration worked well, several respondents expressed insufficient leadership and that collaboration between departments needed to be improved. The NEWS and proACT training were perceived to provide structure and confidence, facilitate clear oral and written communication, and increase patient safety. Level of care influenced the perceived need for NEWS and proACT training. In palliative wards, critical care was expressed as often not applicable. In intensive care and postoperative care, continuously monitoring and acting on critical conditions is a standard procedure and was therefore not an issue. In a context where critical situations occurred less often, the respondents perceived the need as more evident. Several respondents expressed a need for other staff, especially physicians, to have more familiarity with the NEWS and proACT. More training and frequent regular repetition were suggested.

## Discussion

This questionnaire study demonstrated that an interprofessional training intervention that included theory as well as high-fidelity simulation (proACT) [23] improved the capability to identify and manage patients with critical conditions. The healthcare professionals’ competence increased, with a sustained effect over time, whereas no correlation was seen between workplace conditions that support safe care and proACT coverage.

To prevent critical illness and improve the prognosis of the patient, signs of deterioration need to be identified and accompanied by adequate actions, which require frontline staff competence [4, 18, 26]. However, gaining competence is a complex process that is affected by several factors. According to Kolb [27], learning is best achieved through experience and the possibility of combining theory with practice and reflection. Training through simulation can contribute to learning and better performance of practical skills for individual healthcare professionals [28]. Interprofessional simulation-based training also seems to provide the greatest opportunities for improved team functioning [29]. In our study, all professions benefited from increased knowledge from the training, although the knowledge level positively correlated with the length of professional education (highest among physicians and lowest among ANs). Additionally, familiarity and use of the NEWS [10, 11] was increased in the short and long term. The increase was most evident among the physicians, who were the profession that before the training had the lowest familiarity and use of the NEWS. This is of relevance for clinical practice because the physicians are supposed to inquire for the NEWS assessment and use it to seek help from more experienced providers, such as the intensive care unit team.

Increased confidence of the frontline staff to communicate and initiate early treatment could lead to a positive change for critically ill patients and is essential for patient safety [20, 28]. The training interventions increased the reported level of self-confidence to identify, initially treat and communicate a critical condition, with a persistent effect over time. The effect was most prominent among the ANs and significant in some items for the RNs and physicians. This has clinical relevance because ANs are the ones that in Swedish healthcare spend most time bed-side with the patient. The increased level of self-confidence resonates with findings from Hansen et al. [30], who in their evaluation of proACT training in nurse education found increased competence and feelings of security when assessing and managing acutely ill patients. The results also support findings from other interprofessional simulation-based initiatives in different hospital settings [31–34] and programs for healthcare students [35]. Although longitudinal evaluations of training interventions are scant, persistent increased self-confidence is also indicated by Kiessling et al. [31].

Courage among everyone in the team to speak-up if something feels wrong is vital for patient safety [20]. In the present study, the courage to speak-up was significantly higher among proACT-trained ANs compared to the group without such training, but no overall significant effect of the training intervention on the courage was found. This contradicts findings by Connolly et al. [36], who, in their synthesis of learners’ experiences, found that interprofessional simulation increased psychological safety and courage to speak-up to other professions. Robertson et al. [37] conclude from their observation study that power dynamics can sometimes limit learning in discussions and that issues related to power that arise during interprofessional simulations often went unacknowledged, leaving healthcare professionals unprepared to navigate power discrepancies in practice. The respondents’ overall high ratings regarding courage to speak-up, as well as regarding collaboration and workplace climate, do not indicate such issues in the studied setting. However, the role of power dynamics was not explicitly explored and warrants further studies.

Workplace conditions that support safe care, including a workplace climate with reliable collaboration and communication, are crucial for optimal outcomes in complex healthcare [19, 20], which was also emphasized by the respondents in the present study. The findings demonstrate mainly positive perceptions regarding workplace climate and collaboration. However, one-fifth of the respondents would not feel safe if their loved one was cared for in their workplace. A need for further coverage of the proACT principles [23] and NEWS [10, 11] among all staff, and especially among the physicians, was highlighted in the free text answers. Overcrowded wards, insufficient staffing, communication barriers and insufficient access to physicians were presented as sometimes hindering optimal monitoring and adequate management of deteriorating patients. This perception is supported by Smith et al. [38], who proved that staffing level influenced the rate of failure to adequately respond to deranged vital signs. Altogether, the findings confirmed the previously identified issues and need for improvements in the care of patients in general hospital wards [1, 3, 5, 21, 39].

With respect to the complexity of healthcare and the multiple factors influencing a patient´s illness trajectory and outcome, the issue of suboptimal patient safety and resource utilization is inevitably not solved only by uniform routines and training opportunities. No significant correlation was found between workplace conditions that support safe care and the proportion of staff in each care unit who had completed the training intervention. However, interpretation of the effect of the intervention was difficult in this population because of the relatively high ratings for the workplace conditions regardless of the absence of proACT training and by the limited coverage of the intervention, which makes improvement due to an intervention that includes a minority of the staff unlikely. Despite the ambitious initiative of mandatory training for all RNs, ANs and physicians in adult somatic care, the intervention only reached 26.5% of staff, and most units did not have any proACT^−^trained physician at all. Most likely, higher coverage of trained staff is needed to affect the workplace conditions. Ju et al. [40] have distilled twelve guiding principles for interprofessional simulation-based education based on evidence-based guidelines, which plausibly are also relevant for training in healthcare organizations. In these guiding principles, they, among other things, emphasize the importance of equitable distribution across professions, that the scenarios reflect how teams in real life are arranged and that adequate time is allocated for the training (see Table 8). In times of staff constraints, it is a challenge to implement extensive training interventions [41], which was also evident in the present study. In clinical practice, this issue leads to goal conflicts, which need to be considered by decision makers.

**Table 8:** Guiding principles for interprofessional simulation-based education Guiding principles based on Ju et al. [42]

Guiding principles for interprofessional simulation-based education		
	**Principle**	**Description**
1	Equitable distribution	Planning, implementation and learning are done jointly at every level involving all relevant professions. Distribution of roles and responsibilities across professions is balanced
2	Active learning	Activities that promote learners’ cognitive engagement (active participants, not passive bystanders) using strategies such as multiple repetitions, feedback, task variation, or intentional task sequencing
3	Interprofessional competency-based learning objectives	Program has clearly defined learning objectives that focus on competencies for collaborative practice, including interprofessional knowledge, behaviors/skills, and attitudes
4	Interprofessional competency-based assessment	Program assesses learners’ achievement of clearly defined outcomes or benchmarks for competency in collaborative practice, including interprofessional knowledge, behaviors/skills, and attitudes
5	Psychological safety	The program sets up an atmosphere of social acceptance of feedback from all peers, notably through the physical environment and through pre-briefing
6	Repeated and Distributed Practice	There is an opportunity for learners to engage in focused, repeated practice where the intent is skill improvement over a period of time
7	Attention to Differences and Hierarchy	Educators discern and address diversity and differences between groups in educational, professional, and cultural background with sensitivity. They also raise issues related to power inequities between learners
8	Feedback during Debriefing	Debriefing occurs during the simulation session and includes feedback and information on performance provided to learners. Feedback can be provided by facilitators and by peers. Debriefing should be attributed to most experienced educators
9	Sociological fidelity	Scenarios have high levels of social realism and reflect how teams in real life are arranged
10	Program evaluation	Programs are rigorously evaluated as early as possible and involve major stakeholders. The purpose of the evaluation is stated clearly and considers learning outcomes and theoretical perspectives. The results from program evaluation may be disseminated
11	Train facilitators	Educators receive training to understand the ethos, principles and methods for interprofessional simulation-based education. This training focuses on how to develop, deliver, and evaluate the education
12	Institutional support	Institutional policies support educators and health workers to promote interprofessional simulation-based education. They provide adequate financial support (remuneration models, funding streams, incentives for workers to participate), adequate time allocations (regular meetings for interprofessional champions), and adequate space and facilities

To our knowledge, this is the first study assessing an ongoing proACT training intervention in a whole healthcare organization. The findings are of importance in the field since the burden of critical illness is a current and increasing global issue [1, 42, 43]. However, assessing the effect of interprofessional training is a challenge [44]. Although the combination of a cross-sectional survey and a longitudinal of a cohort strengthened the conclusions, the study design limits the ability to draw firm conclusions about whether it was indeed the proACT training that influenced the results. Additionally, other limitations should be considered. First, the study was conducted in three hospitals but within only one healthcare organization. Any generalization of the effects of the intervention should be done with caution, especially to settings with different prerequisites. Second, the response rate of the cross-sectional survey was 29.4%. During the time of the study, the proportion of proACT trained staff in the study setting increased from 13.2% to 26.5%, while 50.2% of the respondents were proACT trained. Hence, staff without proACT training were underrepresented, which may have influenced the results. Third, the response rate in the cohort varied between data collections. Furthermore, few physicians in the training intervention influenced the comparative analysis in the cohort sample. Fourth, low proACT coverage at the end of the study precluded conclusions about eventual effect on the workplace conditions. Last, the measured outcomes have multiple determinants, and the findings were based on a limited knowledge test and self-reported perceptions. Although a positive effect for patients in clinical practice can be presumed, other study designs are needed to investigate such a correlation.

Implications for practice and future research include that capability to identify and manage critical conditions need to be improved. ProACT training can be successful and increase healthcare professionals’ competence, but clear expectations from the management and feasible opportunities to participate may be needed to enable staff of all healthcare professions to take part in such training interventions. Studies of interventions reaching a higher coverage of trained staff are warranted to clarify whether the training can also improve workplace conditions that support safe care. Additionally, studies focusing on the role of power dynamics, and more longitudinal studies are warrant.

## Conclusions

Our findings confirmed previously identified need for improvements in the care of critically ill patients in general hospital wards. One-fifth of the respondents would not feel safe if their loved once was care for in their workplace. Collaboration and workplace climate were perceived to be mainly positive, but a need for improved competence and staffing was emphasized.

The findings support the notion that an interprofessional training intervention that includes theory as well as high-fidelity simulation (proACT) can increase the capability to identify and manage patients with critical conditions. Although the quasi-experimental design cannot with certainty establish causation, proACT seems to overall increase healthcare professionals’ competence, including knowledge, use of NEWS, and self-confidence. However, the proACT coverage was low, and no significant correlation was found between proACT coverage and workplace conditions that support safe care.

### Supplementary Information

Additional file 1. Full distribution of responses regarding familiarity with and use of the NEWS.

Additional file 2. Full distribution of responses regarding levels of self-confidence.

Additional file 3. Full distribution of responses regarding workplace conditions.

## Data Availability

The datasets generated and analyzed during the current study are available from the corresponding author on reasonable request.

## Abbreviations

AN: Assistant Nurse
ANOVA: Univariate Analysis of Variance
COVID-19: The Corona Virus Disease 2019
CRM: Crew Resource Management
CTC: Clinical Training Centre
ICU: Intensive Care Unit
NEWS: National Early Warning Score
RN: Registered Nurse
