# Supplementary material for: Capability to identify and manage critical conditions: effects of an interprofessional training intervention
Source: BMC Med Educ. 2024 May 28;24:584. doi: 10.1186/s12909-024-05567-z (PMC11134908; doi:10.1186/s12909-024-05567-z)
Supplement: Supplementary file 1 — Additional file 1. Full distribution of responses regarding familiarity with and use of the NEWS. [file 12909_2024_5567_MOESM1_ESM.pdf]

Appendix 1 Familiarity and use of the National Early Warning Score (NEWS): full distribution of responses. First, the results from the cross-sectional survey separated responders without proACT versus those who had completed proACT. Second, the results from the three data collections in the longitudinal cohort: before the proACT course (I), the week after (II), and six months later (III).

Familiarity and use of the NEWS routine

Cross-sectional survey data % (n)

		**No proACT**				**Completed proACT**			
		n	No	Unsure	Yes	n	No	Unsure	Yes
I have knowledge of the NEWS [1]	ANs	(n=93)	5.38 (5)	22.6 (21)	72.0 (67)	(n=118)	-	2.54 (3)	97.5 (115)
	RNs	(n=80)	-	22.5 (18)	77.5 (62)	(n=113)	-	8.85 (10)	91.2 (103)
	Physicians	(n=64)	6.25 (4)	37.5 (24)	56.3 (36)	(n=28)	3.57 (1)	14.3 (4)	82.1 (23)
	Total	(n=237)	3.80 (9)	26.6 (63)	69.6 (165)	(n=259)	0.39 (1)	6.56 (17)	93.1 (241)
			Never	Some-times	Daily		Never	Some-times	Daily
I use the NEWS	ANs	(n=93)	16.1 (15)	53.8 (50)	30.1 (28)	(n=118)	0.85 (1)	33.9 (40)	65.3 (77)
	RNs	(n=80)	10.0 (8)	53.8 (43)	36.3 (29)	(n=113)	3.54 (4)	35.4 (40)	61.1 (69)
	Physicians	(n=64)	15.6 (10)	51.6 (33)	32.8 (21)	(n=28)	7.14 (2)	42.9 (12)	50.0 (14)
	Total	(n=237)	13.9 (33)	53.2 (126)	32.9 (78)	(n=259)	2.70 (7)	35.5 (92)	61.8 (160)

Longitudinal cohort data % (n)

		**I - Before proACT**				**II - Week after proACT**				**III - Six month later**			
		n	No	Unsure	Yes	n	No	Unsure	Yes	n	No	Unsure	Yes
I have knowledge of the NEWS [1]	ANs	(n=38)	2.6 (1)	18.4 (7)	78.9 (30)	(n=37)	-	-	100 (37)	(n=32)	-	6.3 (2)	93.8 (30)
	RNs	(n=36)	5.6 (2)	22.2 (8)	72.2 (26)	(n=40)	-	7.5 (3)	92.5 (37)	(n=30)	-	10.0 (3)	90.0 (27)
	Physicians	(n=14)	-	50.0 (7)	50.0 (7)	(n=10)	-	10.0 (1)	90.0 (9)	(n=11)	9.1 (1)	9.1 (1)	81.8 (9)
	Total	(n=88)	3.4 (3)	25.0 (22)	71.6 (63)	(n=87)	-	4.6 (4)	95.4 (83)	(n=73)	1.4 (1)	8.2 (6)	90.4 (66)
			Never	Some-times	Daily		Never	Some-times	Daily		Never	Some-times	Daily
I use the NEWS	ANs	(n=38)	10.5 (4)	55.3 (21)	34.2 (13)	(n=37)	2.7 (1)	54.1 (20)	43.2 (16)	(n=32)	-	40.6 (13)	59.4 (19)
	RNs	(n=36)	8.3 (3)	50.0 (18)	41.7 (15)	(n=40)	10.0 (4)	27.5 (11)	62.5 (25)	(n=30)	10.0 (3)	36.7 (11)	53.3 (16)
	Physicians	(n=14)	28.6 (4)	57.1 (8)	14.3 (2)	(n=10)	10.0 (1)	50.0 (5)	40.0 (4)	(n=11)	9.1 (1)	36.4 (4)	54.5 (6)
	Total	(n=88)	12.5 (11)	53.4(47)	34.1 (30)	(n=87)	6.9 (6)	41.4 (36)	51.7 (45)	(n=73)	5.5 (4)	38.4 (28)	56.2 (41)

Abbreviations: AN, Assistant nurses; RN, Registered nurses; NEWS, National Early Warning Score. Notes: [1)] Response options: "No" = I have previously not heard of the NEWS routine; "Unsure" = I know that the routine exists but is unsure of the content; "Yes" = I know about the routine and are aware of the content and what it means in my work. Variance from 100% is due to rounding.
