# Supplementary material for: Capability to identify and manage critical conditions: effects of an interprofessional training intervention
Source: BMC Med Educ. 2024 May 28;24:584. doi: 10.1186/s12909-024-05567-z (PMC11134908; doi:10.1186/s12909-024-05567-z)
Supplement: Supplementary file 2 — Additional file 2. Full distribution of responses regarding levels of self-confidence. [file 12909_2024_5567_MOESM2_ESM.pdf]

Appendix 2 Level of self-confidence: full distribution of responses. First, the results from the cross-sectional survey separated responders without proACT versus those who had completed proACT. Second, results from the three data collections in the longitudinal cohort: before the proACT course (I), the week after (II), and six months later (III).

Lever of self-confidence – Cross-sectional survey data

		No proACT, % (n)						Completed proACT, % (n)					
		n	Tot. dis.	Partly disagree	Neutral	Partly agree	Totally agree	n	Totally disagree	Partly disagree	Neutral	Partly agree	Totally agree
I am confident to identify signs	ANs	93	1.1 (1)	3.2 (3)	9.7 (9)	61.3 (57)	24.7 (23)	118	-	-	6.8 (8	53.4 (63)	39.8 (47)
	RNs	80	1.3 (1)	1.3 (1)	5.0 (4)	36.3 (29)	56.3 (45)	113	-	1.8 (2)	2.7 (3)	48.7 (55)	46.9 (53)
	Physicians	64	-	1.6 (1)	4.7 (3)	53.1 (34)	40.6 (26)	28	-	-	-	32.1 (9)	67.9 (19)
	Total	237	0.8 (2)	2.1 (5)	6.8 (16)	50.6 (120)	39.7 (94)	259	-	0.8 (2)	4.2 (11)	49.0 (127)	45.9 (119)
I am confident of initial treatment	ANs	93	3.2 (3)	6.5 (6)	25.8 (24)	52.7 (49)	11.8 (11)	118	0.8 (1)	-	13.6 (16)	61.9 (73)	23.7 (28)
	RNs	80	-	3.8 (3)	12.5 (10)	42.5 (34)	41.3 (33)	113	-	0.9 (1)	4.4 (5)	53.1 (60)	41.6 (47)
	Physicians	64	-	1.6 (1)	7.8 (5)	60.9 (39)	29.7 (19)	28	-	-	7.1 (2)	39.3 (11)	53.6 (15)
	Total	237	1.3 (3)	4.2 (10)	16.5 (39)	51.5 (122)	26.6 (93)	259	0.4 (1)	0.4 (1)	8.9 (23)	55.6 (144)	34.7 (90)
I am confident to communicate the condition	ANs	93	3.2 (3)	4.3 (4)	15.1 (14)	46.2 (43)	31.2 (29)	118	-	2.5 (3)	5.9 (7)	44.1 (52)	47.5 (56)
	RNs	80	-	3.8 (3)	5.0 (4)	35.0 (28)	56.3 (45)	113	-	2.7 (3)	2.7 (3)	42.5 (48)	52.2 (59)
	Physicians	64	-	1.6 (1)	6.3 (4)	40.6 (26)	51.6 (33)	28	-		7.1 (2)	32.1 (9)	60.7 (17)
	Total	237	1.3 (3)	3.4 (8)	9.3 (22)	40.9 (97)	45.1 (107)	259	-	2.3 (6)	4.6 (12)	42.1 (109)	51.0 (132)
I am confident to speak-up	ANs	93	2.2 (2)	1.1 (1)	3.2 (3)	40.9 (38)	52.7 (49)	118	-	-	0.8 (1)	29.7 (35)	69.5 (82)
	RNs	80	2.5 (2)	1.3 (1)	3.8 (3)	16.3 (13)	76.3 (61)	113	0.9 (1)	-	2.7 (3)	25.7 (29)	70.8 (80)
	Physicians	64	1.6 (1)	-	7.8 (5)	34.4 (22)	56.3 (36)	28	-	-	-	39.3 (11)	60.7 (17)
	Total	237	2.1 (5)	0.8 (2)	4.6 (11)	30.8 (73)	61.6 (146)	259	0.4 (1)	-	1.5 (4)	29.0 (75)	69.1 (179)

Lever of self-confidence - Longitudinal cohort data

		I - Before proACT, % (n)						II - Week after proACT, % (n)						III - Six months later, % (n)					
		n	Totally disagree	Partly disagree	Neutral	Partly agree	Totally agree	n	Totally disagree	Partly disagree	Neutral	Partly agree	Totally agree	n	Totally disagree	Partly disagree	Neutral	Partly agree	Totally agree
I am confident to identify signs	ANs	38	-	-	31.6 (12)	55.3 (21)	13.2 (5)	37	-	-	5.4 (2)	64.9 (24)	29.7 (11)	32	-	-	6.3 (2)	56.3 (18)	37.5 (12)
	RNs	36	2.8 (1)	8.3 (3)	8.3 (3)	44.4 (16)	36.1 (13)	40	-	-	-	52.5 (21)	47.5 (19)	30	-	3.3 (1)	3.3 (1)	46.7 (14)	46.7 (14)
	Physicians	14	-	-	-	71.4 (10)	28.6 (4)	10	-	-	-	50.0 (5)	50.0 (5)	11	-	-	-	36.4 (4)	63.6 (7)
	Total	88	1.1 (1)	3.4 (3)	17.0 (15)	53.4 (47)	25.0 (22)	87	-	-	2.3 (2)	57.5 (50)	40.2 (35)	73	-	1.4 (1)	4.1 (3)	49.3 (36)	45.2 (33)
I am confident of initial treatment	ANs	38	2.6 (1)	2.6 (1)	36.8 (14)	52.6 (20)	5.3 (2)	37	-	-	10.8 (4)	67.6 (25)	21.6 (8)	32	-	-	9.4 (3)	68.8 (22)	21.9 (7)
	RNs	36	-	11.1 (4)	16.7 (6)	55.6 (20)	16.7 (6)	40	-	-	-	50.0 (20)	50.0 (20)	30	-	-	3.3 (1)	63.3 (19)	33.3 (10)
	Physicians	14	-	-	14.3 (2)	64.3 (9)	21.4 (3)	10	-	-	10.0 (1)	50.0 (5)	40.0 (4)	11	-	-	9.1 (1)	36.4 (4)	54.5 (6)
	Total	88	1.1 (1)	5.7 (5)	25.0 (22)	55.7 (49)	12.5 (11)	87	-	-	5.7 (5)	57.5 (50)	36.8 (32)	73	-	-	6.8 (5)	61.6 (45)	31.5 (23)
I am confident to communicate the condition	ANs	38	-	5.3 (2)	15.8 (6)	52.6 (20)	26.3 (10)	37	-	-	5.4 (2)	45.9 (17)	48.6 (18)	32	-	-	9.4 (3)	37.5 (12)	53.1 (17)
	RNs	36	2.8 (1)	32.8 (1)	19.4 (7)	44.4 (16)	30.6 (11)	40	-	-	-	47.5 (19)	52.5 (21)	30	-	3.3 (1)	6.7 (2)	46.7 (14)	43.3 (13)
	Physicians	14	-	-	14.3 (2)	50.0 (7)	35.7 (5)	10	-	-	10.0 (1)	20.0 (2)	70.0 (7)	11	-	-	9.1 (1)	45.5 (5)	45.5 (5)
	Total	88	1.1 (1)	3.4 (3)	17.0 (15)	48.9 (43)	29.5 (26)	87	-	-	3.4 (3)	43.7 (38)	52.9 (46)	73	-	1.4 (1)	8.2 (6)	42.5 (31)	47.9 (35)
I am confident to speak-up	ANs	38	-	-	2.6 (1)	42.1 (16)	55.3 (21)	37	-	-	5.4 (2)	21.6 (8)	73.0 (27)	32	-	-	-	25.0 (8)	75.0 (24)
	RNs	36	2.8 (1)	-	-	44.4 (16)	52.8 (19)	40	-	-	-	35.0 (14)	65.0 (26)	30	-	-	3.3 (1)	20.0 (6)	76.7 (23)
	Physicians	14	-	-	7.1 (1)	28.6 (4)	64.3 (9)	10	-	-	20.0 (2)	40.0 (4)	40.0 (4)	11	-	-	-	45.5 (5)	54.5 (6)
	Total	88	1.1 (1)	-	2.3 (2)	40.9 (36)	55.7 (49)	87	-	-	4.6 (4)	29.9 (26)	65.5 (57)	73	-	-	1.4 (1)	26.0 (19)	72.6 (53)

AN, Assistant nurses; RN, Registered nurses. Variance from 100% is due to rounding
