# Supplementary material for: Capability to identify and manage critical conditions: effects of an interprofessional training intervention
Source: BMC Med Educ. 2024 May 28;24:584. doi: 10.1186/s12909-024-05567-z (PMC11134908; doi:10.1186/s12909-024-05567-z)
Supplement: Supplementary file 3 — Additional file 3. Full distribution of responses regarding workplace conditions. [file 12909_2024_5567_MOESM3_ESM.pdf]

Appendix 3 Workplace conditions as perceived by the total sample in the cross-sectional survey: full distribution of responses.

Workplace conditions							
**Question**	**Responders**		**Responses % (n)**				
			Very badly	Badly	Neutral	Well	Very well
Collaboration with assistant nurses works	ANs	(n=211)	-	1.90 (4)	1.90 (4)	30.3 (64)	65.9 (139)
	RNs	(n=206)	0.48 (1)	1.46 (3)	6.31 (13)	36.4 (75)	55.3 (114)
	Physicians	(n=98)	2.04 (2)	4.08 (4)	7.1 (7)	49.0 (48)	37.8 (37)
	Total	(n=515)	0.57 (3)	2.14 (11)	4.66 (24)	36.3 (187)	56.3 (290)
Collaboration with registered nurses works	ANs	(n=211)	-	-	1.90 (4)	27.5 (58)	70.6 (149)
	RNs	(n=206)	-	-	1.94 (4)	23.3 (48)	74.8 (154)
	Physicians	(n=98)	1.02 (1)	3.1 (3)	4.08 (4)	42.9 (42)	49.0 (48)
	Total	(n=515)	0.19 (1)	0.57 (3)	2.33 (12)	28.7 (148)	68.2 (351)
Collaboration with physicians works	ANs	(n=211)	1.90 (4)	2.84 (6)	11.8 (25)	41.2 (87)	42.2 (89)
	RNs	(n=206)	0.97 (2)	4.44 (9)	10.2 (21)	46.6 (96)	37.9 (78)
	Physicians	(n=98)	3.06 (3)	-	6.12 (6)	36.7 (36)	54.1 (53)
	Total	(n=515)	1.74 (9)	2.91 (15)	10.1 (52)	42.5 (219)	42.7 (220)
			Totally disagree	Partly disagree	Neutral	Partly agree	Totally agree
I meet positive response when I need help	ANs	(n=211)	0.47 (1)	0.95 (2)	3.32 (7)	32.2 (68)	63.0 (133)
	RNs	(n=206)	1.46 (3)	1.46 (3)	4.37 (9)	26.7 (55)	66.0 (136)
	Physicians	(n=98)	-	1.02 (1)	9.18 (9)	40.8 (40)	49.0 (48)
	Total	(n=515)	0.78 (4)	1.16 (6)	4.85 (25)	31.7 (163)	61.6 (317)
Most staff use the NEWS routine	ANs	(n=211)	2.37 (5)	2.84 (6)	19.0 (40)	43.6 (92)	32.2 (68)
	RNs	(n=206)	5.34 (11)	8.74 (18)	28.6 (59)	36.9 (76)	20.4 (42)
	Physicians	(n=98)	3.06 (3)	10.2 (10)	29.6 (29)	45.9 (45)	11.2 (11)
	Total	(n=515)	3.69 (19)	6.60 (34)	24.9 (128)	41.4 (213)	23.5 (121)
I would feel safe if a loved one was cared for at my workplace	ANs	(n=211)	1.42 (3)	3.79 (8)	12.3 (26)	33.6 (71)	48.8 (103)
	RNs	(n=206)	1.46 (3)	3.40 (7)	15.0 (31)	34.0 (70)	46.1 (95)
	Physicians	(n=98)	3.06 (3)	5.10 (5)	14.3 (14)	40.8 (40)	36.7 (36)
	Total	(n=515)	1.75 (9)	3.88 (20)	13.8 (71)	35.1 (181)	45.4 (234)

Abbreviations: AN, Assistant nurses; RN, Registered nurses; NEWS, National Early Warning Score
